# Effects of neutral polysaccharide from *Platycodon grandiflorum* on high-fat diet-induced obesity *via* the regulation of gut microbiota and metabolites

**DOI:** 10.3389/fendo.2023.1078593

**Published:** 2023-01-26

**Authors:** Jing Song, Qin liu, Mengqi Hao, Xiaohu Zhai, Juan Chen

**Affiliations:** ^1^ College of pharmacy, Anhui University of Chinese Medicine, Hefei, Anhui, China; ^2^ Yunnan Key Laboratory for Fungal Diversity and Green Development, Kunming, Yunnan, China; ^3^ MOE-Anhui Joint Collaborative Innovation Center for Quality Improvement of Anhui Genuine Chinese Medicinal Materials, Hefei, Anhui, China; ^4^ Anhui Province Key Laboratory of Pharmaceutical Preparation Technology and Application, Hefei, Anhui, China

**Keywords:** platycodon grandiflorum polysaccharide, obesity, lipids metabolism, inflammation, gut microbiota, metabolites

## Abstract

The obesity epidemic has become a global problem with far-reaching health and economic impact. Despite the numerous therapeutic efficacies of *Platycodon grandiflorum*, its role in modulating obesity-related metabolic disorders has not been clarified. In this study, a purified neutral polysaccharide, PGNP, was obtained from *Platycodon grandiflorum*. Based on methylation and NMR analyses, PGNP was found to be composed of 2,1-β-D-Fruf residues ending with a (1→2)-bonded α-D-Glcp. The protective effects of PGNP on high-fat HFD-induced obesity were assessed. According to our results, PGNP effectively alleviated the signs of metabolic syndrome, as demonstrated by reductions in body weight, hepatic steatosis, lipid profile, inflammatory response, and insulin resistance in obese mice. Under PGNP treatment, intestinal histomorphology and the tight junction protein, ZO-1, were well maintained. To elucidate the underlying mechanism, 16S rRNA gene sequencing and LC-MS were employed to assess the positive influence of PGNP on the gut microbiota and metabolites. PGNP effectively increased species diversity of gut microbiota and reversed the HFD-induced imbalance in the gut microbiota by decreasing the Firmicutes to Bacteroidetes ratio. The abundance of *Bacteroides* and *Blautia* were increased after PGNP treatment, while the relative abundance of *Rikenella*, *Helicobacter* were reduced. Furthermore, PGNP notably influenced the levels of microbial metabolites, including the increased levels of cholic and gamma-linolenic acid. Overall, PGNP might be a potential supplement for the regulation of gut microbiota and metabolites, further affecting obesity.

## Graphical Abstract

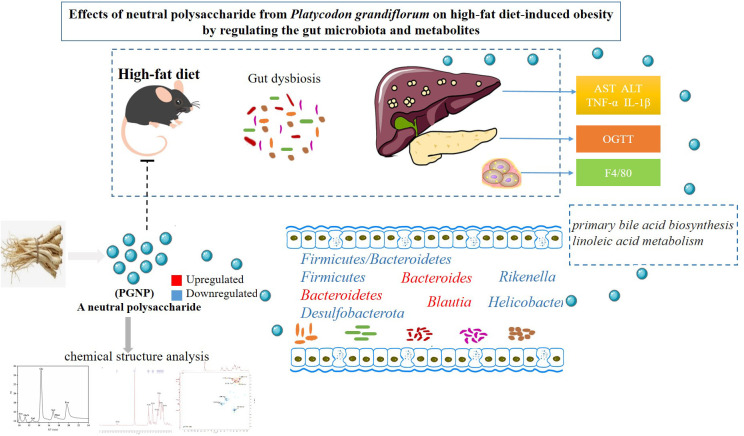

## 1 Introduction

Dietary treatments for obesity have become a central focus of research (1, 2). Severe obesity is known to be a major factor for cardiovascular disease (3), metabolic disease, and cancer (4). A high-fat diet may lead to increased production of oxidative stress in the liver, which may trigger lipid accumulation in the liver, leading to hepatic steatosis or many other diseases (5). Obesity always comes with higher levels of inflammatory factors, such as tumor necrosis factor-α (TNF-α) and interleukin-6 (IL-6) (6). However, the pathological and molecular mechanisms of obesity development have not been investigated. Based on emerging research, the intestinal microbiome, as an environmental factor, plays an important role in the event and advancement of adiposity (7, 8). The gut contains a large reservoir of symbiotic microbes that contribute to nutrient acquisition and energy regulation (9, 10). In recent years, growing evidence has demonstrated that obesity reduces the diversity and richness of the intestinal microbiome (11–14). Research progress on gut microbiota and its correlation with obesity has gradually changed people’s understanding and the investigation of adiposity (15).

Currently, few drugs can be used to reduce body weight. In addition to increasing the burden on the liver and kidneys, drugs may cause myopathy and rhabdomyolysis (16, 17). Certain plant ingredients, such as polysaccharides from *Ganoderma lucidum* (18), *Sarcodon aspratus* polysaccharides (19), and *Raphanus sativus* polysaccharides (20), have demonstrated the potential to improve obesity. Indigestible polysaccharides (dietary fibre) are regularly utilised as prebiotics, such as natural inulin-type fructans, which are digested by gut microbiota and exhibit prebiotic abilities to promote probiotic growth.

*Platycodon grandiflorum* is a daily dietary material and traditional oriental medicine that is commonly used in China, South Korea, and other Asian countries. Polysaccharides are the main components responsible for biological functions. According to previous studies, polysaccharides of *Platycodon grandiflorum* have anti- oxidative stress (21) and immunomodulatory activities (22). Further, a recent study revealed that *Platycodon grandiflorum* polysaccharides have a certain balancing effect on the intestinal flora after exposure to PM2.5 (23). However, only few studies have been conducted on polysaccharides isolated from *Platycodon grandiflorum* for the amelioration of dyslipidaemia *via* intestinal flora modulation in obese mice.

In the current study, a neutral polysaccharide was obtained from *Platycodon grandiflorum*, and its chemical structure was revealed using Fourier transform-infrared spectroscopy (FT-IR), high-performance liquid chromatography (HPLC), methylation analysis, and 1D and 2D nuclear magnetic resonance (NMR) spectroscopy. Biochemical and pathological methods were also used to determine the effects of PGNP on body weight, lipid metabolism and inflammation in HFD-induced obese mice. Notably, PGNP had an enormous influence on gut dysbiosis in mice based on 16S rRNA and LC-MS based microbiological and metabolite profile analysis. Thus, our findings highlight the positive role of PGNP in the control of HFD-induced obesity by regulating gut microbiota and metabolites.

## 2 Materials and methods (Experimental subjects)

### 2.1 Isolation and purification of PGNP

**Figure 1:**
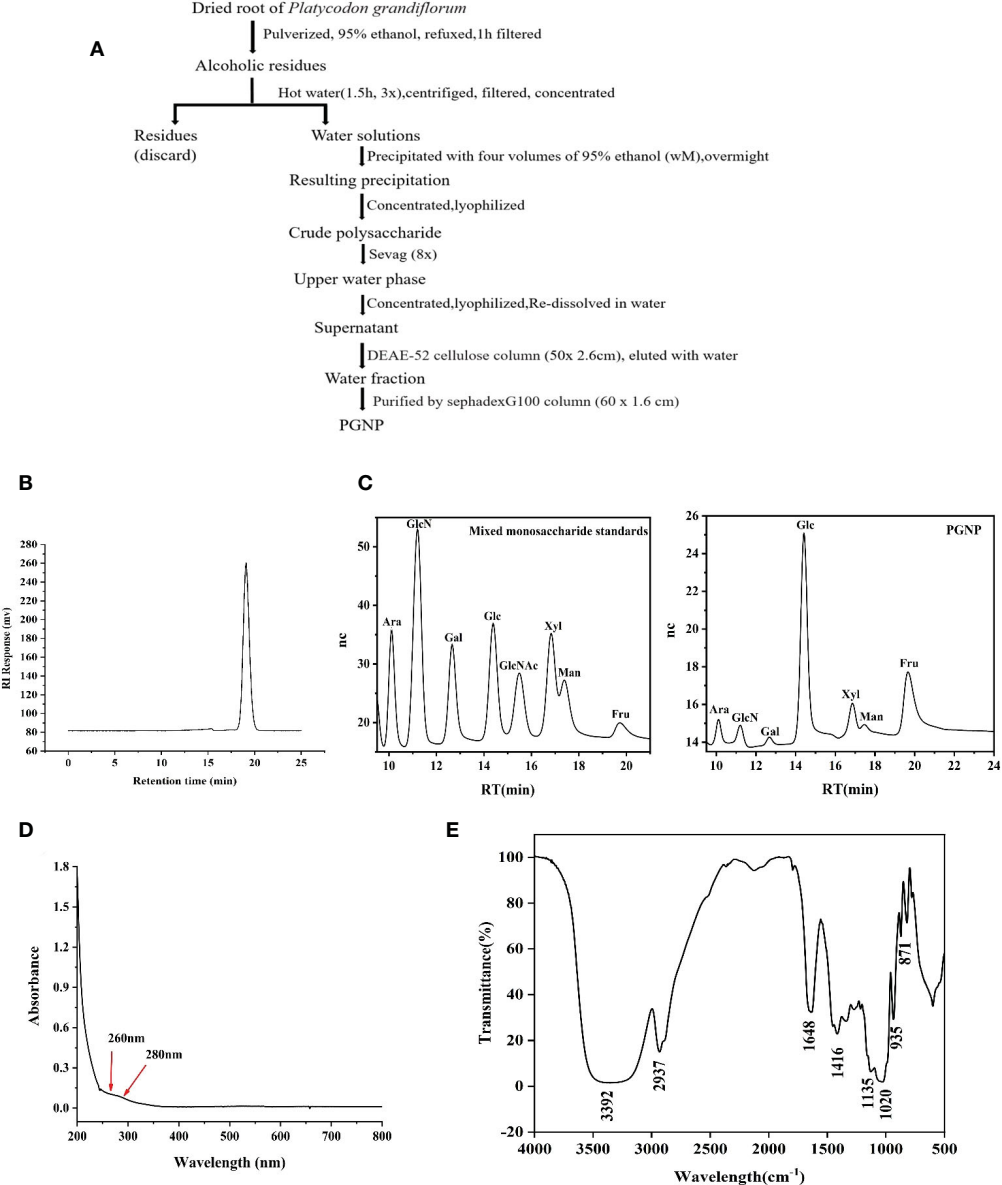
Extraction, purification, and characterization of polysaccharide PGNP from *Platycodon grandiflorum*. Flow chart for the extraction and purification of PGNP **(A)**. HPLC chromatogram of PGNP demonstrating its molecular weight distribution **(B)**. Monosaccharide composition of PGNP identified by the IC chromatogram method. IC chromatograms of standard monosaccharides and PGNP **(C)**. Ultraviolet full wavelength scanning of PGNP **(D)**. FT-IR spectrum of PGNP **(E)**.

Dried roots of *Platycodon grandiflorum* (Jacq.) A. DC. were purchased from Hefei Mandi New Pharmaceutical Co., Ltd., and identified and stored at the College of Pharmacy, Anhui University of Chinese Medicine. *Platycodon grandiflorum* was used as a raw material to extract polysaccharides *via* hot water extraction and 85% ethanol/water precipitation. The extract was deproteinised using the Sevag method, which was repeated several times. The polysaccharide-rich fraction was dissolved in distilled water (1:100), filtered with a 0.45 μm filter, and purified using cellulose DEAE-52 and Sephadex G-100 columns (60 × 1.6 cm). Finally, the purified neutral *Platycodon grandiflorum* (PGNP) was obtained for further identification (**Figure 1A**). The phenol-sulfuric acid method (24, 25) was used to determine the carbohydrate content of the PGNP. With ascorbic acid as the control, the scavenging ability of PGNP on DPPH radical scavenging was determined.

### 2.2 Chemical identification of PGNP

The FT-IR spectra were recorded using an FT-IR spectrometer (Thermo Electron, USA) in the range of 4000–400 cm^−1^ (26). Thereafter, ion chromatography (IC) was performed to detect the monosaccharide composition of polysaccharides. Briefly, the dried PGNP (20 mg) was hydrolysed with 6 M HCL at 105°C for 8 h. The filtrate was eluted with distilled water and 15 mM NaOHC,15 mM NaOH, and 100 mM NaOAC at a flow rate of 0.3 mL/min. The monosaccharide standards were purchased from Sigma-Aldrich Chemical Co., Inc. The homogeneity and molecular weight of PGNP were detected using HPLC on an Agilent 1260 instrument (Agilent, USA) (27). The PGNP was methylated using previously reported methods (26). PGNP (60 mg) was dissolved in 1.0 mL D_2_O. The ^1^H NMR, ^13^C NMR, and two-dimensional NMR (including COSY, HMBC, and HSQC) spectra were recorded on a Bruker 600 MHz NMR spectrometer.

### 2.3 Anti-obesity effects *in vivo*

**Figure 2:**
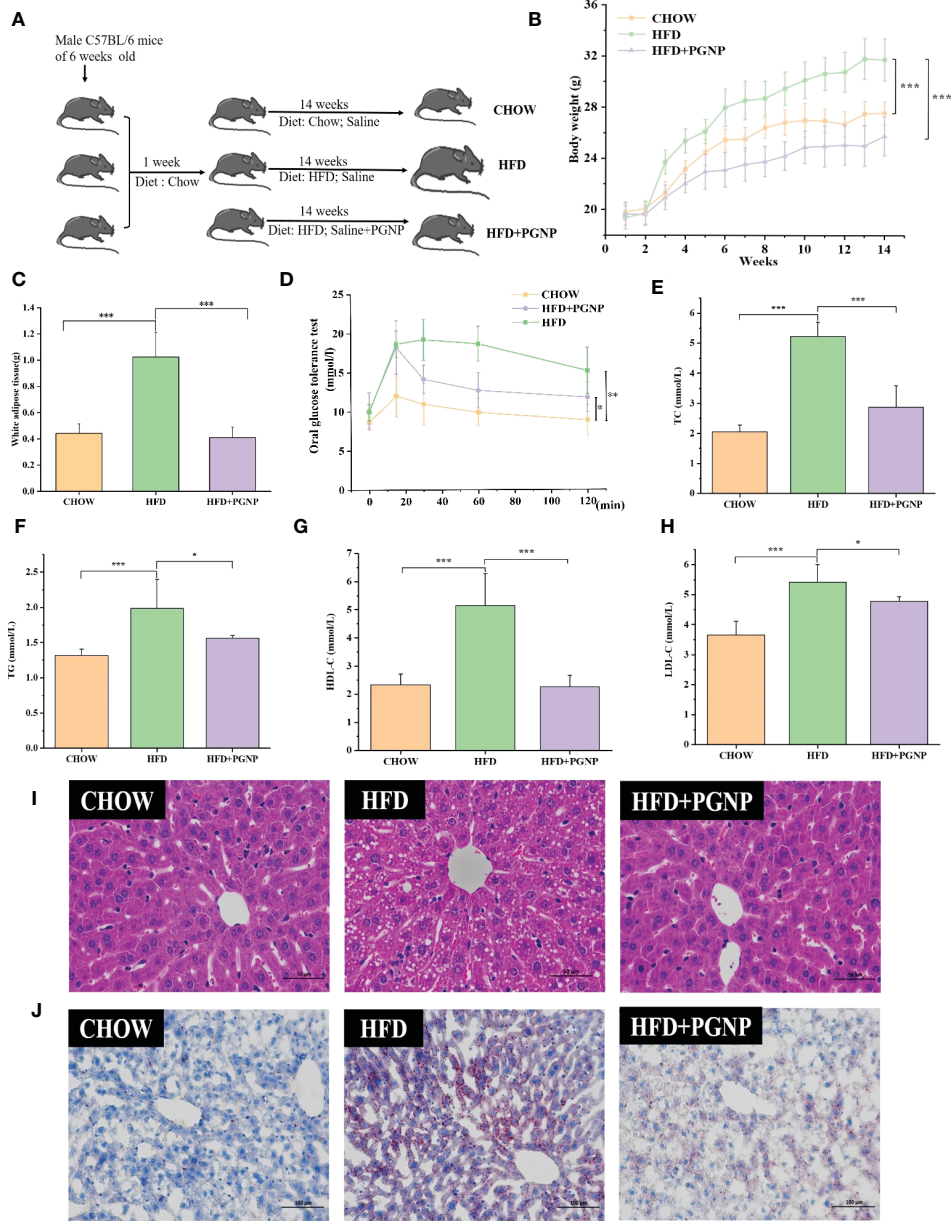
Effects of PGNP on body weight gain and fat accumulation in HFD-fed mice. Schematic representation of animal experiment process **(A)** Body weight, two-way anova coupled with Turkey’s multiple-comparison *post hoc* test was used, and *P*<0.001 was determined and shown at week 14 **(B)** Epididymal white adipose tissue **(C)** Oral glucose tolerance test (OGTT) **(D)** TC levels in serum **(E)** TG levels in serum **(F)** HDL-C levels in serum **(G)**; LDL-C levels in serum **(H)** Liver tissue sections histologically stained with H&E and Oil Red O **(I, J)** ^***^ *P* < 0.001, ^**^ *P* < 0.01, ^*^ *P* < 0.05.

This animal trial was conducted under the supervision of the Ethics Committee of the Anhui University of Chinese Medicine, Hefei, China. Eight-week-old male C57BL/6 mice were supplied by Anhui Medical University (Hefei, China) (Reg. No. SCXK (Wan) 2019-0004). The timeline of animal administration is outlined in **Figure 2A**. After a 1-week acclimation period, mice were randomly divided into three groups: one group was fed a chow diet (D12450B, 10% energy derived from fat, Research Diets, Inc.), while the remaining two groups were fed an HFD (D12492 60% of energy from fat, Research Diets, Inc.) with or without PGNP (300 mg/kg/day) *via* oral gavage. Each cage contained three animals and was housed under pathogen-free conditions with temperature control (25−28°C) and light-dark cycles. Body weight and fodder intake were recorded weekly throughout the experimental period. At 14 weeks, the main organs of animals and their serum were collected after killing with carbon dioxide. The epididymal white adipose tissue (EWAT) was collected and weighed, and the caecal contents of five mice in each group were collected in sterile cryovials, frozen in liquid nitrogen, and stored at -80°C for the analysis of gut microbiota.

### 2.4 Histopathological and biochemical analyses

Liver and colon tissues were fixed in 4% paraformaldehyde and embedded in paraffin, as previously described (19). All the biochemical parameters in serum were measured according to the manufacturer’s instructions. The serum collected from all mice was analysed for TC, total cholesterol, TG, triglyceride, LDL-C, low-density lipoprotein cholesterol, AST, aspartate aminotransferase, ALT, and alanine aminotransferase levels using commercial kits (Nanjing Jiancheng Institute of Bioengineering, Nanjing, China). Serum interleukin-1β (IL-1β) and tumour necrosis factor-α (TNF-α) levels were measured using commercial ELISA kits (Dogesce, China).

### 2.5 Oral glucose tolerance test (OGTT)

The impaired glucose tolerance range was detected after HFD feeding and PGNP intervention for 12 weeks. Mice in each group fasted for 12 h before testing. Subsequently, 2 g/kg D-glucose was administered to mice *via* oral gavage (28). The tail blood glucose levels of mice were monitored at 0, 30, 60, and 120 min using glucose kits. The blood glucose-time correlation curves were plotted, and the area defined by the glucose concentration curve at the top and the x-axis (time) at the bottom was determined using SPSS 11.5 software (SPSS Inc., Chicago, IL, USA).

### 2.6 Immunohistochemistry (IHC) assay

The EWAT and colon were placed in a fresh 10% paraformaldehyde solution, wrapped in paraffin blocks, and cut into thin sections (4 µm) for occult IHC. The sections were then dewaxed with xylene followed by graded ethanol, washed three times with PBS, and treated with 10% foetal bovine serum at 37°C for 30 min. The EWAT was then incubated with the anti-F4/80 (Wuhan Servicebio Technology Co., Ltd, Wuhan, China) primary antibody, while the colon was incubated with the ZO-1(Wuhan Servicebio Technology Co., Ltd, Wuhan, China) primary antibody. After three washes with PBS, the cells were incubated with HRP-labelled secondary antibody for 30 min. Finally, all slides were counterstained with haematoxylin.

### 2.7 Gut microbiota analysis

Bacterial DNA was separated from the caecal contents using a DNeasy PowerSoil kit (Qiagen, Hilden, Germany). The V3–V4 region of 16S rRNA was amplified using the universal primers, 343F and 798R. 16S rRNA gene amplicon sequencing of faecal samples was performed by OE Biotech Co. Ltd. on a MiSeq platform (Illumina, San Diego, CA, USA). The sequence was processed according to a previous method and stored in FASTQ format (29). The raw data were processed using the QIIME2 software package (version 2020.11). Clustering sequences within a 97% similarity threshold for OTUs using Vsearch software. All representative reads were annotated and blasted against Silva database (Version 138) using q2-feature-classifier with the default parameters. QIIME2 software was used for alpha and beta diversity analysis. Alpha diversity was estimated using the Simpson diversity index. The unweighted Unifrac distance matrix performed by R package was used for unweighted Unifrac Principal coordinates analysis (PCoA) to estimate the beta diversity. Then the R package was used to analyze the significant differences between different groups using ANOVA and T test.

### 2.8 Untargeted faecal metabolomics analysis

The intestinal contents were analysed, as previously described (30). The analytical instrument used in this study was an LC/MS system composed of an ACQUITY UPLC I-Class PLUS ultra-high-performance liquid phase tandem QE plus high-resolution mass spectrometer. The LC-MS analysis data of each sample were collected in negative ion mode and positive ion mode. Spearman correlation analysis was performed using R version 3.6.2 to evaluate the correlations between bacterial taxa and metabolomics and biochemical index.

### 2.9 Statistical analysis

*In vivo* experimental data are presented as mean ± SD of body weight gain, while other parameters are presented as mean ± SEM. Differences between groups were analysed using one-way ANOVA or repeated measures two-way ANOVA and Student’s t-test. The outcomes for alpha diversity (Simpson index) are expressed as median ± interquartile range (IQR). The differences between groups were based on Anosim analysis. A significant difference between the data was indicated by *p*< 0.05. Statistical analysis was performed using SPSS 11.5 software (SPSS Inc., Chicago, IL, USA). Raw LC-MS data were processed using Progenesis QI V2.3 software (Nonlinear, Kinetics, Newcastle, UK) for baseline filtering, peak identification, integration, retention time correction, alignment, and peak normalisation.

## 3 Results

### 3.1 Preparation and preliminary characterization of PGNP

After a series of processes (**Figure 1A**), a neutral polysaccharide was successfully purified from *P. grandiflorum* (PGNP). Based on the calibration curve with standard dextran, PGNP was a purified fraction with an average molecular weight of approximately 13.6 kDa. PGNP was characterised by a single symmetrical peak in HPLC (**Figure 1B**). The sugar content of PGNP measured using the phenol-sulfuric acid method was 92%. The PGNP had obvious DPPH free radical scavenging abilities within a certain concentration range in a concentration-dependent manner but were still lower than the positive control (vitamin C) (**Figure S1F**). As shown in **Figure 1C**, PGNP was mainly composed of glucose and fructose, with molar percentages of 10.9% and 84.1%, respectively. As the foremost monosaccharide component of PGNP was fructose, the bone chain may consist of fructose residues. PGNP contained almost no protein, and no obvious absorption peak was found at 280 nm in the UV spectrum (**Figure 1D**). **Figure 1E** shows the infrared spectrum of PGNP. The broad characteristic strong peak near 3384 cm^-1^ was attributed to the OH stretching vibration of the polysaccharide (31), while the peak at 2937 cm^-1^ was attributed to the C-H stretching vibration (32). PGNP might have a β-configuration glycosidic bond due to the bands at 935 and 871 cm^-1^ (33, 34).

#### 3.1.1 Methylation analysis

Methylation analysis of PGNP was carried out to determine the type of linkage between fructosyl residues. Fructose can be converted to mannitol and glucitol under reducing conditions (35). **Table 1** shows the results of the methylation analysis, which suggested that PGNP is an inulin-type fructan containing a β-D-(2→1)-linked linear backbone.

**Table 1:** Linkages of PGNP determined *via* GC–MS methylation analysis.

Partially O-methylated alditol acetates	Diagnostic fragments	Deduced residues
2,5-Di-O-acetyl-(2-deuterio)-1,3,4,6-tetra-O-methyl glucitol	205,161,129,117,101,87	t-Fruf
2,3,4,6-Me4-Glcp	207,162,129,101,87	t-Glcp
1,2,5-Tri-O-acetyl-(2-deuterio)-3,4,6-tri-O-methyl mannitol	233,207,189,161,129,101,87	(2→1)-Fruf

#### 3.1.2 NMR analysis

The structure of PGNP was further explained by ^1^H-NMR, ^13^C-NMR, and two-dimensional NMR (COSY, HSQC, and HMBC) (**Table 2**). A minor peak was detected in the anomeric region (5.40-5.46 ppm) in **Figure S1A**, indicating the existence of an anomeric proton in Glc (36). Notably, the signal at 92.56 ppm in the anomeric carbon region is based on C-1 of α-D-Glcp. Meanwhile, the signals assigned to the C-2 of β-D-Fruf were observed at 102.99–103.60 ppm (**Figure S1B**). Based on the H-H COSY (**Figure S1C**), the generation of signals at δH 4.27/4.11 and δH 4.11/3.87 is due to the H3-H4 and H4-H5 of fructose residues, respectively. Of note, a weak cross-peak, denoted as Glcp, appeared in the HSQC spectra between δ 5.44 and 92.42 ppm (**Figure S1D**). Glycosyl residue sequences were determined using HMBC experiments. As shown in **Figure S1E**, the extraordinary cross-peak between H-1 and C-2 of the residue manifested a (2→1)-linkage between fructosyl residues, which aligned with an inulin-type structure.

**Table 2:** Assignments of the ^1^H and ^13^C NMR spectra of PGNP based on an analysis of the COSY, HSQC, and HMBC spectra.

Glycosyl residues	Proton	Carbon
β-D-Fruf-2,1	H1a (δ 3.73), H1b (δ 3.93) H2 (-) H3 (δ4.27) H4 (δ4.12) H5 (δ3.88) H6a (δ3.78), H6b (δ3.85)	C1 (δ60.77) C2 (δ103.25) C3 (δ77.08) C4 (δ74.38) C5 (δ81.15) C6 (δ62.15)
t-α-D-Glcp	H1 (δ5.46)	C1 (δ92.56)

### 3.2 PGNP plays a critical role in body weight, blood plasma lipid concentrations, and dextrose tolerance in mice fed a high-fat diet

To investigate the modulation of PGNP on the disordered profile of glycolipid metabolism, obese mice were administered PGNP (300 mg/kg) for 14 weeks. As expected, mice fed the HFD had significantly higher body weights than those fed the low-fat diet (**Figure 2B**, *P*<0.001). Notably, PGNP treatment attenuated weight gain in the HFD group (31.734 ± 1.64 *vs* 25.75 ± 1.52g, *P*<0.001). During the intervention period, no significant differences in the mean daily food intake were found (**Figure S2A**), which suggested that the effect of PGNP on body weight and fat parameters was not based on decreased fodder utilisation. The average daily food intake (kcal) levels in the HFD group were significantly higher than the CHOW group (*P*<0.001, **Figure S2B**).

**Figure 3:**
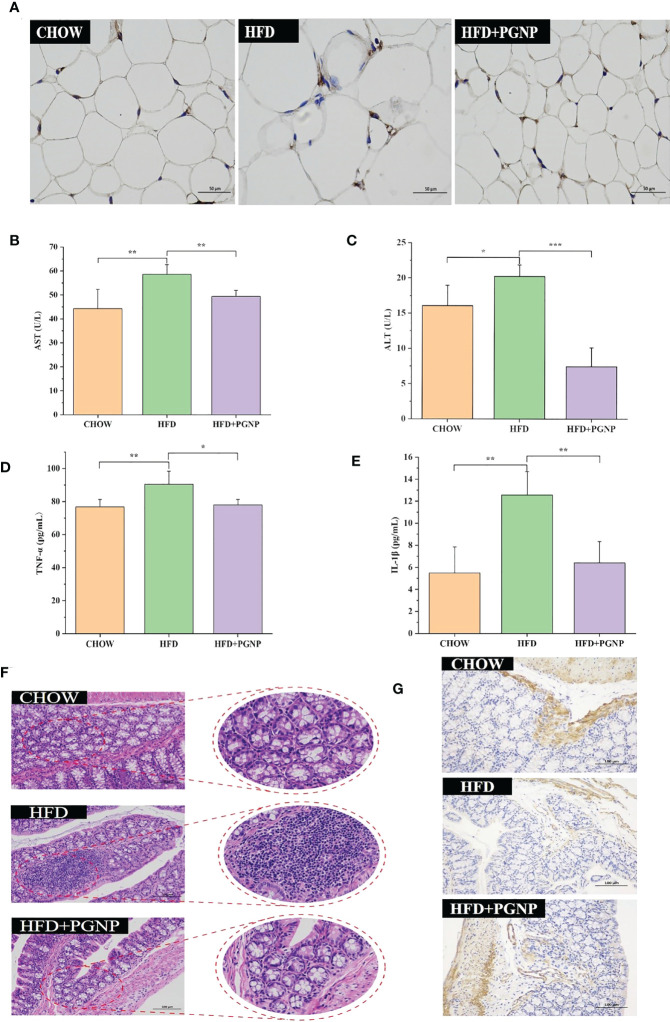
Effects of PGNP on lipid metabolism and chronic low-grade inflammation. Immunohistochemical staining of F4/80 expression **(A)** AST levels in serum **(B)** ALT levels in serum **(C)** TNF-α levels in serum **(D)** IL-1β levels in serum **(E)** Colon tissue sections histologically stained with H&E **(F)** Immunohistochemical staining of ZO-1 expression **(G)**. ^***^ *P* < 0.001, ^**^ *P* < 0.01, ^*^ *P* < 0.05.

As depicted in **Figure 2C**, EWAT weight markedly increased after 14 weeks in the HFD-fed group, while PGNP supplementation reversed this change (*P*<0.001 *vs* HFD group). Histological analysis confirmed that the HFD group had larger adipocytes than the CHOW group. Further, the HFD + PGNP group showed a significant decrease in the mean size of adipocytes (**Figure S2D**, *P*<0.001), which was similar to that of the CHOW group (**Figure 3A**).

The serum lipid product content was analysed in HFD-fed mice. Serum TG, TC, and LDL-C levels in the CHOW group were lower than those in the HFD group (*P*<0.05, *P*<0.001, respectively). Furthermore, PGNP treatment significantly reduced the levels of TC and TG in the serum of HFD-fed mice (**Figures 2E, F**, *P*< 0.001, *P*< 0.05). We assessed the impact of PGNP on glucose metabolism after 12 weeks of HFD feeding. During the OGTT, fasting glucose levels in the HFD group were approximately 15.17 ± 2.9 mmol/L. After PGNP treatment, obese mice developed good glucose tolerance with a smoother line (**Figure 2D**). Similarly, the glucose concentration profile of the PGNP-treated group was reduced compared to that of the HFD group (**Figure S2C**, *P*<0.01). These results suggest that PGNP was remarkably beneficial to body weight gain, impaired glucose tolerance, and disordered lipid profile induced by HFD feeding.

### 3.3 Impacts of PGNP on liver function, inflammation, and metabolic disorders

Arresting hepatic steatosis and hypertrophy of hepatocytes were observed in the HFD group through haematoxylin and eosin and Oil Red O staining. Hepatic cells with critical ballooning degeneration and higher hepatic lipid droplets were observed in the HFD group (**Figures 2I, J**). After 14 weeks, PGNP suppressed the abnormal accumulation of lipid droplets in the hepatic tissue (**Figures 2I, J**). Furthermore, PGNP significantly reduced ALT and AST levels, which were increased by the HFD (**Figures 3B, C**).

Alterations in glycolipid metabolism are often accompanied by chronic low-grade inflammation, which is a malignant process. Thus, the accumulation of triglycerides in adipose tissue accelerates the production of the pro-inflammatory factors, IL-1β and TNF-α. Conversely, a strong inflammatory response contributes to adiposity. In this study, PGNP supplementation markedly reduced the serum expression of IL-1β and TNF-α compared to HFD alone (**Figures 3D, E**). Meanwhile, immunohistochemical staining revealed the F4/80 of macrophages in adipose tissue (brown cells; **Figure 3A**). Evidently, the HFD group had more brown areas than the PGNP group (**Figure S2F**). In summary, PGNP had positive ameliorating effects on HFD-related metabolic characteristics, which were revealed by the prevention of liver function and adipose inflammation.

### 3.4 Histological observation of the colon

Colonic histological examination was performed using haematoxylin and eosin staining. The HFD group displayed inflammation-related histopathological changes. Inflammation in the colon usually manifests as abundant red blood cells, lymphocytes, and loosely arranged intestinal glands. Significant differences in intestinal morphology were observed between the CHOW and HFD groups, whereas no significant differences were observed between the PGNP and CHOW groups (**Figure 3F**). Zo-1 expression (brown) in colon tissue was determined by IHC. The emergence of Zo-1 in the HFD group was markedly lower than that in the PGNP group (**Figures 3G**, **S2E**, *P*<0.001).

### 3.5 PGNP improves HFD-induced intestinal dysbacteria

**Figure 4:**
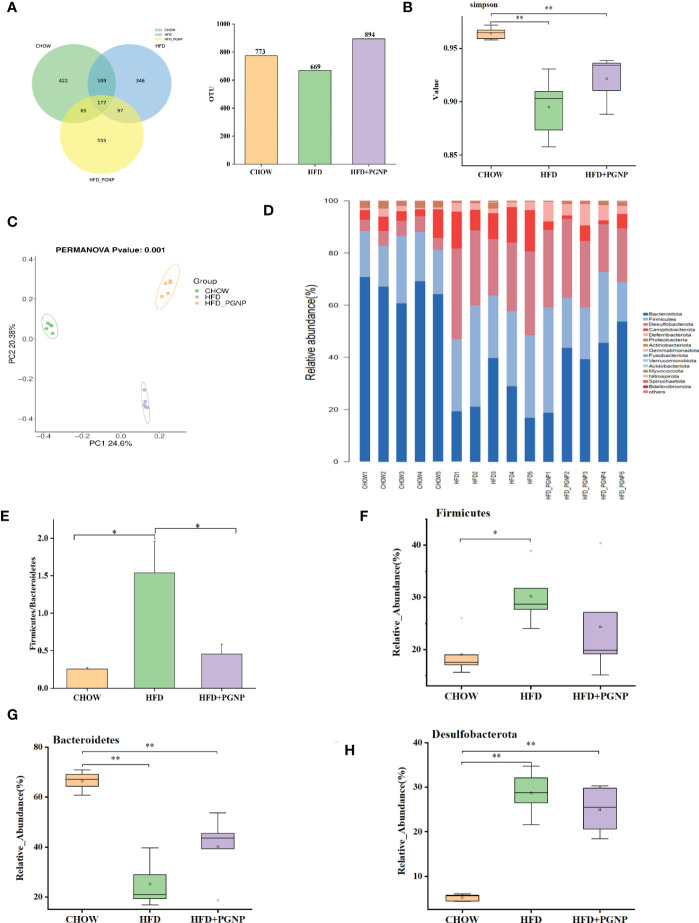
Effects of PGNP on gut microbial dysbiosis in HFD-fed mice (*n*=5). Venn graph of the OTUs from gut microbiota of the Chow (green), HFD (blue), and HFD + PGNP(yellow) groups **(A)**. Simpson index **(B)**. Weighted UniFrac PCoA of gut microbiota based on the OTU data of three groups **(C)**. The component of gut microbiota at the phylum level **(D)**. Firmicutes/Bacteroidetes abundance ratio in faecal microbiota in Chow, HFD, and HFD + PGNP groups **(E)**. Relative abundance of Firmicutes **(F)**. Relative abundance of Bacteroidetes **(G)**. Relative abundance of Desulfobacterota **(H)**. ^**^ *P* < 0.01, ^*^ *P* < 0.05.

To evaluate the regulatory role of PGNP on gut bacteria, the IlluminaMiQ platform was used to analyse the bacterial 16S rRNA in the variable region V3-V4 of the caecal samples based on pyrosequencing. After the exclusion of unqualified sequences, high-throughput barcode sequencing yielded 1,083,465 high-quality sequences from 15 samples. There were 773, 669, and 894 mice in the Chow, HFD, and HFD + PGNP groups, respectively (**Figure 4A**). However, the Chow and HFD groups only had 422 unique OTUs and 364 unique OTUs, respectively. The 555 unique OTUs in the HFD + PGNP group were investigated (**Figure 4A**). The Simpson index of the PGNP group was significantly higher than that of the HFD group, indicating that PGNP can effectively increase the diversity of the microflora (**Figure 4B**). Clustering of the study participants was performed using PCoA based on weighted UniFrac distances. As shown in **Figure 4C**, PGNP markedly changed the intestinal flora composition.

To further determine the distinct bacterial communities among the groups, we analysed the similarity of bacterial taxa at the phylum level. Accordingly, the top 15 most abundant phyla were assessed. The HFD group had a higher relative abundance of Firmicutes, Desulfobacterota, and Campilobacterota phyla. However, the relative abundance of the Bacteroidetes phylum decreased considerably in the HFD group. Surprisingly, PGNP reversed the above changes in relative abundance (**Figure 4D**). The increased Firmicutes/Bacteroidetes (**Figure 4E**) abundance ratio positively correlates with the obesity phenotype (37). Notably, PGNP treatment led to a reduction in the Firmicutes/Bacteroidetes abundance ratio.

**Figure 5:**
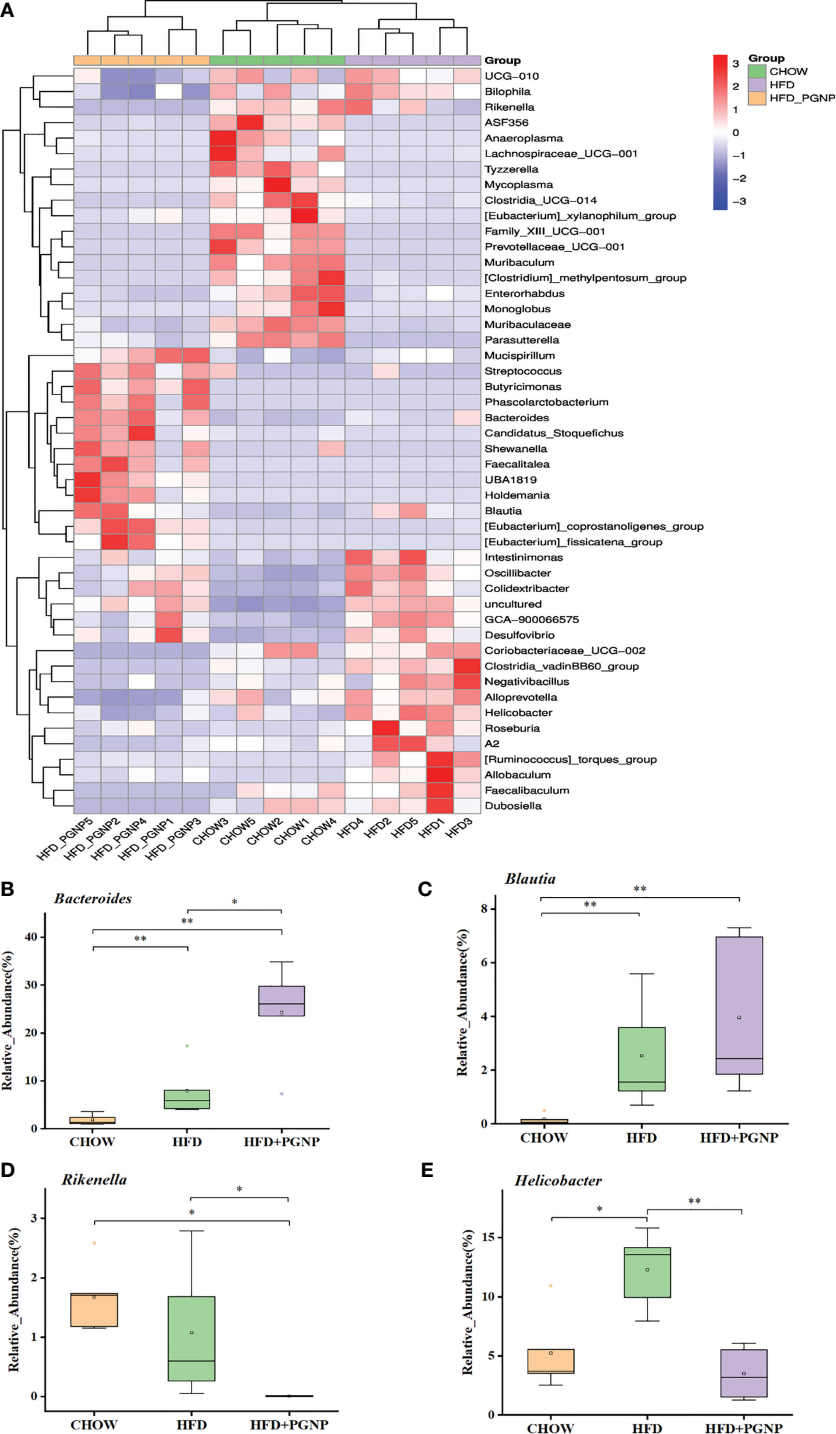
Heatmap of microbial distributions at the genus level for each sample. Blocks in red and blue denote high and low z-score values of OTU sizes, respectively **(A)**. Effects of PGNP on the relative abundance of *Bacteroides* **(B)**, *Bualtia* **(C)**, *Rikenella* **(D)**, and *Helicobacter* **(E)** in HFD-fed mice. ^**^ *P* < 0.01, ^*^ *P* < 0.05.

We proceeded to investigate the composition of the gut microbiota at the genus level to further evaluate its structure. The abundance of *Bacteroides* (**Figure 5B**, *p* < 0.05) and *Blautia* (**Figure 5C**) were improved after PGNP treatment, which was decreased by the consumption of an HFD; conversely, for *Rikenella*, the PGNP group exhibited lower levels than the HFD group (**Figure 5D**, *p* < 0.05). The level of *Helicobacter* was significantly elevated in the HFD group (*p* < 0.05 *vs* CHOW group, **Figure 4E**). Intriguingly, after PGNP treatment, the levels of the above bacteria in the CHOW group were similar to those in the HFD group (**Figure 5C**).

### 3.6 PGNP adjusts metabolites in HFD-fed mice

**Figure 6:**
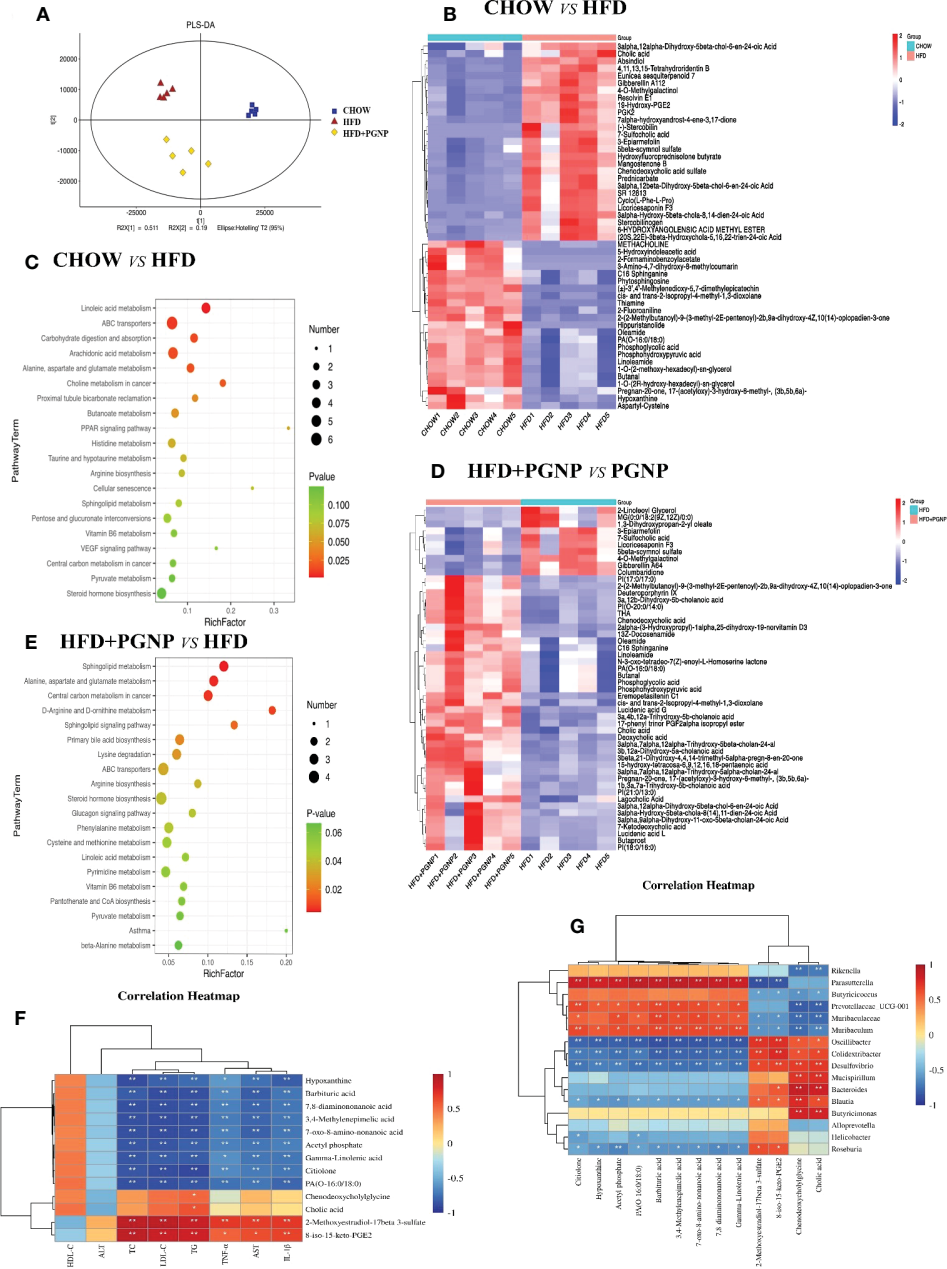
Alterations of the metabolite levels in samples after HFD or HFD + PGNP treatment. PLS-DA of metabolites in the CHOW, HFD, and HFD+PGNP groups **(A)**. Heatmap of the altered metabolites in CHOW and HFD **(B)**. KEGG enriched pathways of the altered metabolites in CHOW and HFD **(C)**. Heatmap of the altered metabolites in HFD+PGNP and HFD **(D)**. KEGG enriched pathways of the altered metabolites in HFD+PGNP and HFD **(E)**. Correlation of metabolites and gut microbiota **(F)**. Correlation of metabolites and pharmacodynamic parameters **(G)**. *P < 0.05; **P < 0.01.

Altered gut microbiota composition has a documented relationship with adiposity and metabolic syndromes. Intestinal microorganisms metabolise the intestinal contents and produce different metabolites. To explore the relationship between intestinal microbes and metabolites, the intestinal contents were analysed using LC-MS. Partial least-squares discriminant analysis (PLS-DA) clearly revealed predictive and descriptive modelling. Further, the PLS-DA results revealed that the metabolite profiles in the three groups were markedly modified (**Figure 6A**).

The differentially expressed metabolites were identified by the VIP value of OPLS-DA (VIP>1) combined with the Student’s T test (**Figure 6A**, *P*<0.05). A notable difference in metabolites was found between the CHOW and HFD groups. A total of 824 metabolites were altered when the CHOW and HFD groups were compared. Of these metabolites, 333 were upregulated and 491 were downregulated (**Figure S2G**). Furthermore, the crucial altered metabolic pathways of metabolic syndrome, linoleic acid metabolism, PPAR signalling pathway, steroid hormone biosynthesis, and dehydroepiandrosterone sulphate (DHEAS), were available for metabolic enrichment and pathway analysis based on a database search (**Figures 6B, C**).

A comparison of the HFD+PGNP and HFD groups revealed 513 differential metabolites, with 227 upregulated and 286 downregulated metabolites (**Figure S2H**). **Figure 6E** shows a schematic of the affected metabolic pathways, for example, linoleic acid metabolism, d-arginine and d-ornithine metabolism, and primary bile acid biosynthesis. PGNP treatment significantly altered the HFD profile after 14 weeks, significantly increasing the levels of both saturated fatty acids and bile acids (**Figure 6D**).

To clarify the exact role of PGNP in improving metabolic disorders, the causal links between the host gut microbiota and metabolites were assessed using Spearman correlation analysis. Based on our results, the gut microbiota community structures were closely related to the gut metabolites and pharmacodynamic parameter profiles. Herein 16 microbiota taxa were identified at the genus level. Further, pharmacodynamic parameters *in vivo* were positively or negatively associated with differential metabolites (**Figures 6F, G**). Among them, HDL-C was positively associated, whereas seven other pharmacodynamic parameters were negatively associated with gamma-linolenic acid.

## 4 Discussion

Dietary changes have been verified as the most prominent shapers of gut microbiota composition, which reflects metabolite profiles and major physiological phenotypes. Accordingly, examining the protective effects of food-derived polysaccharides on obesity is important. To our knowledge, this is the first study to reveal that a neutral polysaccharide isolated from *Platycodon grandiflorum* could suppress obesity-related symptoms by altering the composition of gut microbiota and metabolites.

*Platycodon grandiflorum* is a common edible plant and a traditional Chinese medicine that belongs to the *Campanulaceae* family and is mainly distributed in Northeast Asia. Polysaccharides have been reported to be the main components responsible for their biological functions. Through isolation and purification, a neutral polysaccharide was obtained from *Platycodon grandiflorum*, which is mainly composed of fructose and glucose in a molar ratio of 84.1% to 10.9%. Methylation and NMR analyses revealed that PGNP has a linear backbone composed of 2,1-β-D-Fruf residues, ending with a (1→2)-bonded α-D-Glcp. From the above analysis, the main monosaccharide component of PGNP is fructose, which is consistent with the results of previous studies (38). Fructans are indigestible and no absorbed carbohydrates are fermented by the bacterial flora upon reaching the colon. Generally, adiposity is strongly related to long-term excessive intake of energy-dense foods. In our study, PGNP effectively reduced the typical parameters of obesity in HFD-fed mice for 14 weeks, indicating that PGNP can increase fat consumption. Consistently, the serum levels of TG, TC, LDL-C, and blood glucose in HFD-fed mice decreased sharply after PGNP supplementation. Our findings indicated that PGNP ameliorates obesity-induced weight gain, fat accumulation, and insulin resistance.

At a broader level, lipid droplet formation in hepatocytes and hepatocyte steatosis are the most typical pathological changes observed in obese patients (39). In this study, inflammatory infiltration and lipid droplet accumulation were observed in the liver tissues of obese mice. However, PGNP reduced the formation of hepatic lipid droplets in HFD-fed mice (**Figures 2J**, **3A**). AST and ALT levels are important parameters for assessing acute or chronic liver injury. PGNP significantly reduced ALT and AST levels, which were increased by HFD (**Figures 3B, C**). These results imply that in lipid metabolism disorder, PGNP has beneficial effects on the improvement of liver tissue function.

Chronic inflammation is deeply involved in metabolic disorders, such as obesity (40). PGNP was found to markedly reduce the levels of pro-inflammatory cytokines in serum, such as TNF-α and IL-1β (**Figures 3D, E**). IHC was used to assess local inflammation in the adipose tissues. HFD mice had higher levels of macrophages in the liver and adipose tissue than normal-fed mice. Macrophage levels decreased in HFD-fed mice after PGNP treatment. Thus, PGNP attenuates the inflammatory response and reduces macrophage infiltration in HFD-fed mice. Numerous studies have confirmed that a long-term high-energy diet could disrupt the intestinal barrier, which increases LPS entering the blood circulation and causes chronic inflammation, adiposity, and insulin resistance in the body (41). ZO-1 is an important protein that maintains the intestinal barrier function. PGNP increased ZO-1 protein expression in our study, which was beneficial in improving chronic inflammation (**Figure 3G**).

The gut microbiota is well known to be associated with obesity (42). Obesity generally involves a lower level of intestinal Bacteroidetes and a higher level of Firmicutes, indicating that these major phyla may play a decisive role in obesity-induced inflammation. Of note, existing findings suggest that the diversity and richness of intestinal flora are reduced in obese humans (11). Numerous studies have shown that inulin-type fructan has beneficial prebiotic efficacy and excellent performance in regulating intestinal flora. The activity of PGNP, an inulin-type fructan, has been extensively studied in the intervention of intestinal flora in obese mice.

In this study, based on the results of pyrosequencing analysis, PGNP significantly increased the diversity of the gut microbiota. Correspondingly, PGNP effectively reversed the decrease in the abundance of Bacteroidetes and the increase in Firmicutes caused by HFD. On one hand, an extensive analysis supported the conclusion that Firmicutes can markedly ferment and metabolize carbohydrates and lipids, thereby contributing to the development of obesity. Notably, increased Firmicutes produces more lipopolysaccharide and deoxycholic acid, which enter the liver through the hepatic portal vein, leading to liver inflammation (43, 44). On the other hand, Bacteroidetes mainly inhabit the distal part of the intestine and are involved in the fermentation of dietary fibres, such as cellulose, hemicellulose, β-glucan, etc (45).

At the phylum level, the ratio of Firmicutes to Bacteroidetes is a crucial indicator of the intestinal microbial population in obesity (46). Accordingly, the ratio of Firmicutes/Bacteroidetes was reduced by PGNP in the HFD-fed group. Of note, HFD had a 6-fold larger ratio of Firmicutes/Bacteroidetes than CHOW. Moreover, PGNP treatment markedly reduced (70%) the Firmicutes/Bacteroidetes ratio. In addition, PGNP significantly reduced the abundance of Desulfobacterota, which may liberate LPS into the intestine to cause an inflammatory response and disrupt intestinal energy metabolism (47). Overall, the improvement of PGNP on adiposity may be related to intestinal flora.

At the genus level, the levels of *Bacteroides* and *Blautia* in the PGNP group were higher than those in the HFD group. *Bacteroides* can break down polysaccharides, and the products released by *Bacteroides* are beneficial for the host (48). Moreover, an increase in *Bacteroidetes* is significantly associated with weight loss (46). *Blautia* can mediate beneficial anti-inflammatory effects (49). In summary, the ameliorating effect of PGNP on obesity might be related to the elevation of *Bacteroides* and *Blautia*. Remarkably, a reduction in *Helicobacter* was observed in the PGNP group, whereas an increase was observed in the HFD group. *Helicobacter* enhance the intestinal immune system, prompting the body to secrete inflammatory factors, thereby promoting insulin resistance, together with the synergistic effect of intestinal flora, which is predisposing factor for obesity (50, 51).

Gut microbiota generate numerous metabolites that are beneficial to human health. In recent years, SCFAs have been among the most intensively studied multifarious metabolites produced by gut microbiota. Few studies have investigated the benefits of fatty acids in the host. For instance, pentadecanoic acid may have led to significant declines in the TC and LDL-C levels (52). Differential metabolites from the major metabolic pathways were identified using KEGG. Thus, the levels of both γ-linolenate and eicosanoids in CHOW increased compared to those in HFD. Eicosanoids and γ-linolenate are long chain fatty acids. Based on this study, γ-linolenate supplementation effectively suppressed weight loss (53). Such findings suggest that linoleic acid has long-term benefits in preventing type 2 diabetes. Eicosanoids activate PPARγ receptors to promote adipocyte differentiation and lipid metabolism. Intriguingly, of the discrepant metabolites between the CHOW and HFD groups, the thiamine content was found to be higher in the HFD group. According to prior studies, increased thiamine intake reduces the risk of metabolic syndrome (54).

The beneficial effect of PGNP on HFD-induced adiposity is mainly reflected in the regulation of metabolites in two aspects: long-chain fatty acids and bile acid metabolism. On one hand, compared to the HFD group, eicosanoids must be increased after PGNP treatment. Inflammation is affected by fatty acids *via* various mechanisms ranging from the cell membrane to the nucleus. Eicosanoids produced from arachidonic acid are well known to play a critical role in inflammation (55). In contrast, the content of cholic acid was altered by HFD and reversed by PGNP. A large body of evidence indicates that bile acid has a favourable effect on obesity, type 2 diabetes, dyslipidaemia, and nonalcoholic fatty liver disease (56, 57). Cholic acid might strengthen glucose metabolism and energy expenditure through the bile acid-signalling pathway.

Intestinal metabolites co-produced by the host and the intestinal flora play a crucial role in maintaining host health. To identify the relationships between differential metabolites and the beneficial effects of PGNP, the metabolite profiles and metabolic pathway variations were analysed. The “harmful” bacteria *rikenalla* and *helicobacter* were negatively correlated with cholic acid, while “beneficial” bacteria had no correlation with cholic acid. *Blautia* has a clear relationship with changes in the bile acid pool and enhances some important secondary bile acids (58). Notably, changes in these bile acids were associated with changes in glucose and lipid metabolism, particularly in glucose and high-density lipoprotein cholesterol levels, suggesting that these secondary bile acids, formed by specific members of the gut microbiota, may have an effect.

The relevance of metabolites and pharmacodynamic parameters revealed that γ-linolenic acid weakened the serum levels of AST, ALT, TC, TG, LDL-C, TNF-α, and IL-1β. Gamma-Linolenic acid plays an essential role in metabolic disorders and is an anti-inflammatory metabolite that inhibits leukotriene B4 biosynthesis (59). Herein, gamma-linolenic acid was significantly negatively correlated with *Helicobacter*. Our findings indicate that PGNP enhanced these two metabolites, which were reduced by HFD intake, indicating the beneficial effect of PGNP on adiposity. Other metabolites that may be involved in the improvement of high-fat diet-induced obesity by PGNP were also found. Based on the above analysis, PGNP has a moderating effect on obesity-induced gut microbiota imbalance and metabolic disorders.

As mentioned above, this is the first study to highlight the protective effects of a neutral polysaccharide from *Platycodon grandiflorum* on HFD-induced obesity through animal experiments. Our preliminary results suggest that PGNP effectively alleviated obesity-related parameters, as demonstrated by reductions in body weight gain, hepatic steatosis, lipid profile, inflammatory response, and insulin resistance in obese mice. Furthermore, the abundance of Bacteroidetes, *Bacteroides*, and *Blautia* improved after PGNP treatment. Primary bile acid biosynthesis and linoleic acid metabolism were found in the metabolite pathway enrichment analysis, indicating the significance of these two pathways in the amelioration of metabolic disorders.

Altogether, the current study highlights that PGNP has beneficial effects on improving HFD-induced metabolic disorders by regulating intestinal metabolism and gut microbial, it has not been explored in depth and there is a lack of intestinal flora transplantation verification. Carbohydrates are diverse bio-macromolecules with highly complex structures. In this study, PGNP is a novel purified polysaccharide. The interaction between the structural features of PGNP and specific gut microbiota would be explored *via* carbohydrate enzymes. The intestinal flora of mice should be transplanted into HFD-induced obesity in mice after administration of PGNP to observe whether it can treat obesity, which may become a new breakthrough in the treatment of obesity. Our findings provide critical new evidence of the underlying mechanisms of PGNP in treating metabolic disorders at both gut microbiota and metabolism levels.

## Data availability statement

The raw data supporting the conclusions of this article will be made available by the authors, without undue reservation.

## Ethics statement

The animal study was reviewed and approved by Ethics Review Committee of Anhui University of Traditional Chinese Medicine.

## Author contributions

JS: Methodology, writing – original draft, data curation. QL: Methodology. MH: Methodology and investigation. XZ: Formal analysis. JC: Conceptualisation, writing – review and editing, funding acquisition, project administration, and investigation. JS and QL contributed equally to this study. All authors contributed to the article and approved the submitted version.

## Supplementary material

Supplementary Figure 1 1H NMR **(A)**, 13C NMR **(B)**, COSY **(C)**, HSQC **(D)**, and HMBC spectra of PGNP **(E)**, Scavenging effect of PGNP and Vitamin C (Vc) at different concentrations on DPPH radicals **(F)**.

Supplementary Figure 2 Average daily intake(g) **(A)**; Average daily intake(kcal) **(B)**; The area of OGTT **(C)**; Mean fat area (50 μm) **(D)**; Relative expression of ZO-1 **(E)**; Relative expression of F4/80 **(F)**; Volcano plot of altered metabolites with VIP > 1 and *P* < 0.05 in a two-tailed, unpaired Student’s *t*-test between two groups, Chow and HFD groups **(G)**; HFD and HFD + PGNP groups **(H)**. ****P* < 0.001, ***P* < 0.01.
