# Supplementary material for: Effects of neutral polysaccharide from Platycodon grandiflorum on high-fat diet-induced obesity via the regulation of gut microbiota and metabolites
Source: Front Endocrinol (Lausanne). 2023 Jan 26;14:1078593. doi: 10.3389/fendo.2023.1078593 (PMC9908743; doi:10.3389/fendo.2023.1078593)
Supplement: Supplementary Figure 1 — 1H NMR (A), 13C NMR (B), COSY (C), HSQC (D), and HMBC spectra of PGNP (E), Scavenging effect of PGNP and Vitamin C (Vc) at different concentrations on DPPH radicals (F). [file DataSheet_1.docx]

Supplementary Material

# Figure S1 1H NMR (A), 13C NMR (B), COSY (C), HSQC (D), and HMBC spectra of PGNP (E), Scavenging effect of PGNP and Vitamin C (Vc) at different concentrations on DPPH radicals (F).

# Figure S2 Average daily intake(g) (A); Average daily intake(kcal) (B); The area of OGTT (C); Mean fat area (50 μm) (D); Relative expression of ZO-1 (E); Relative expression of F4/80 (F); Volcano plot of altered metabolites with VIP > 1 and P < 0.05 in a two-tailed, unpaired Student’s t-test between two groups, Chow and HFD groups (G); HFD and HFD + PGNP groups (H). ^***^*P* < 0.001, ^**^*P* < 0.01.

**Figure S1**


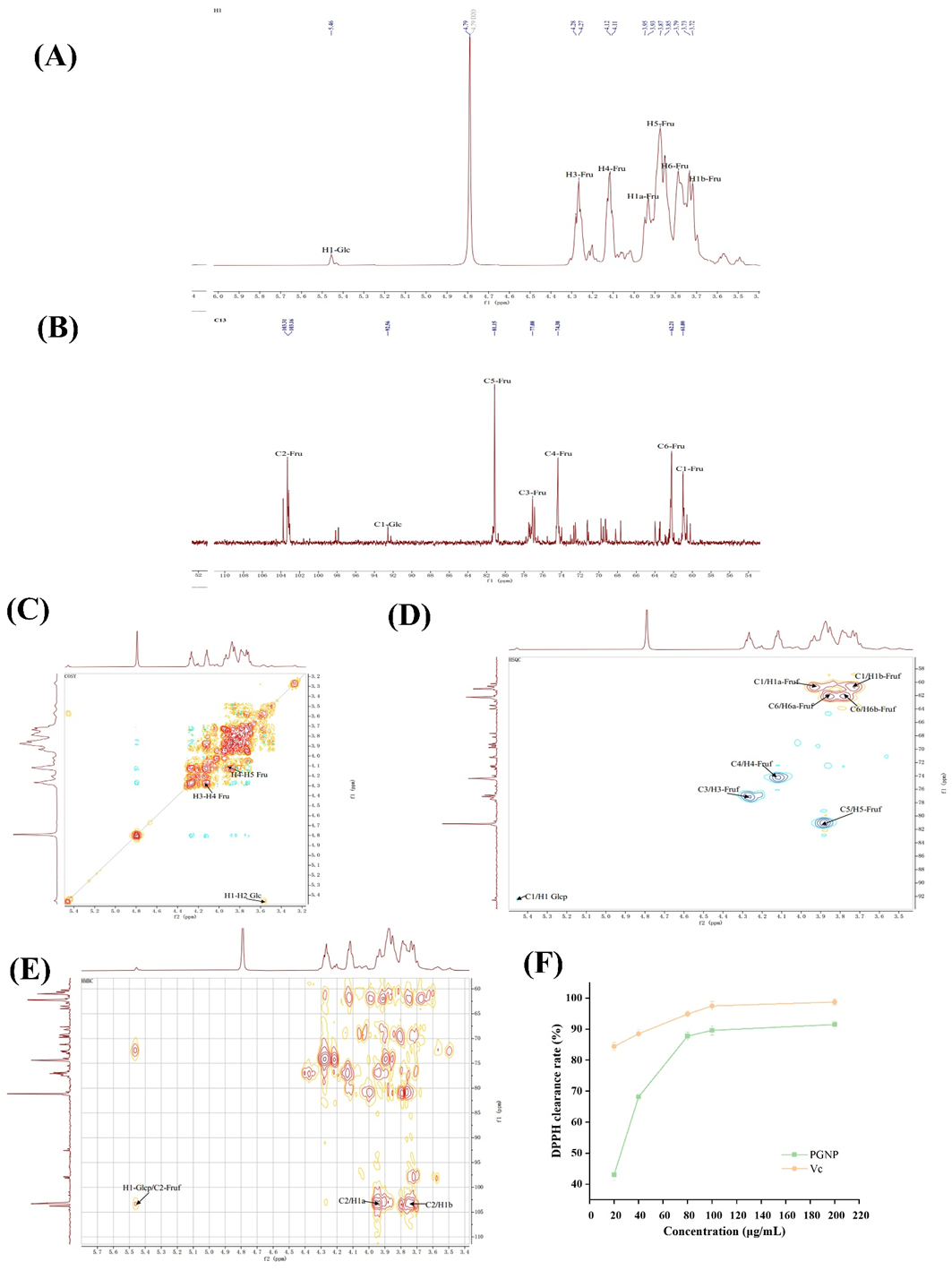


**Figure S2**

**
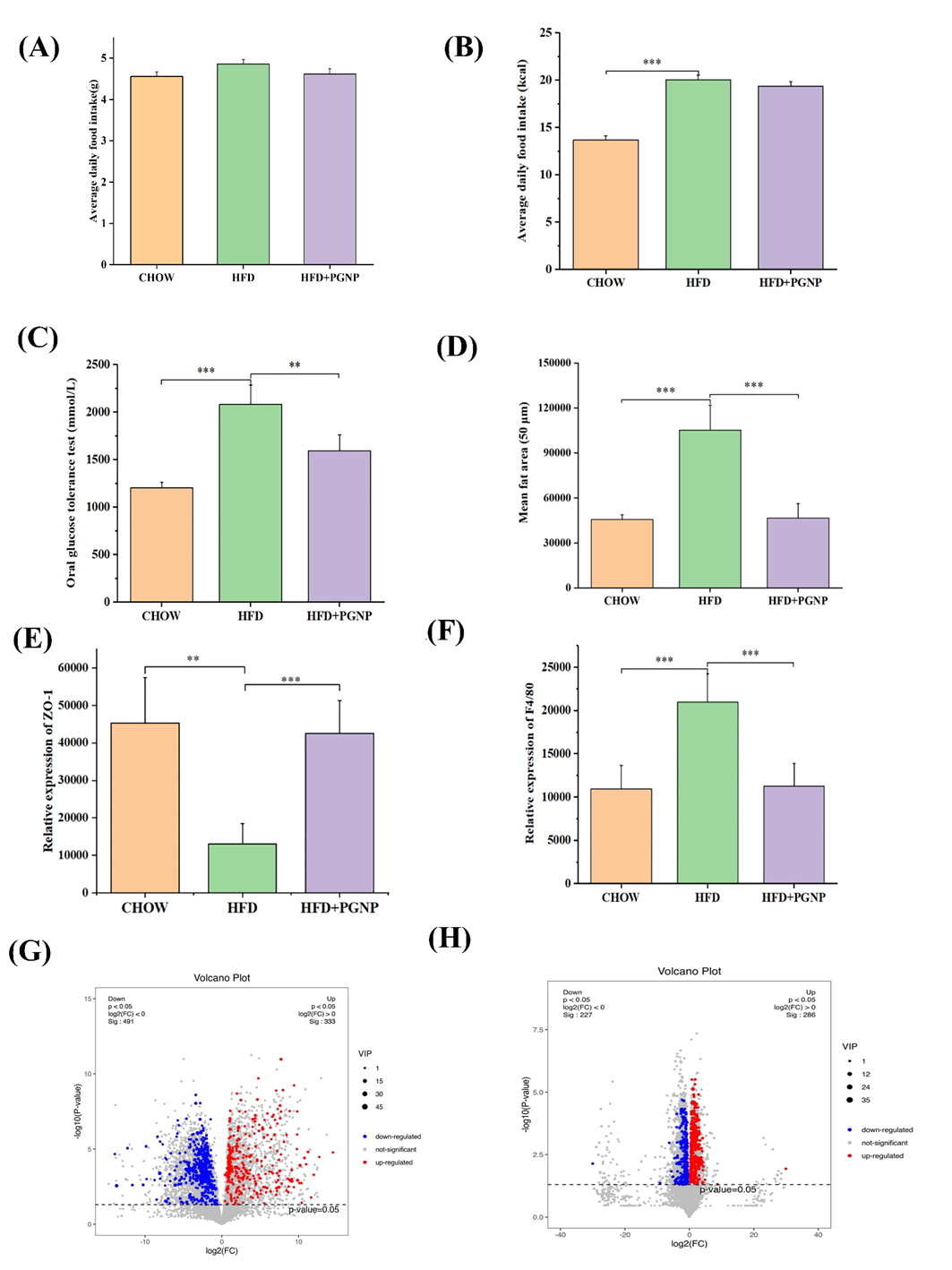
**
